# Optimal linear ensemble of binary classifiers

**DOI:** 10.1093/bioadv/vbae093

**Published:** 2024-06-25

**Authors:** Mehmet Eren Ahsen, Robert Vogel, Gustavo Stolovitzky

**Affiliations:** Department of Business Administration, University of Illinois at Urbana-Champaign, Champaign, IL, 61820, United States; Department of Biomedical and Translational Sciences, University of Illinois at Urbana-Champaign, Urbana, IL, 61801, United States; Thomas J. Watson Research Center, IBM, New York, NY 10598, United States; Department of Integrated Structural and Computational Biology, Scripps Research, La Jolla, CA 92037, United States; Thomas J. Watson Research Center, IBM, New York, NY 10598, United States

## Abstract

**Motivation:**

The integration of vast, complex biological data with computational models offers profound insights and predictive accuracy. Yet, such models face challenges: poor generalization and limited labeled data.

**Results:**

To overcome these difficulties in binary classification tasks, we developed the Method for Optimal Classification by Aggregation (MOCA) algorithm, which addresses the problem of generalization by virtue of being an ensemble learning method and can be used in problems with limited or no labeled data. We developed both an unsupervised (uMOCA) and a supervised (sMOCA) variant of MOCA. For uMOCA, we show how to infer the MOCA weights in an unsupervised way, which are optimal under the assumption of class-conditioned independent classifier predictions. When it is possible to use labels, sMOCA uses empirically computed MOCA weights. We demonstrate the performance of uMOCA and sMOCA using simulated data as well as actual data previously used in Dialogue on Reverse Engineering and Methods (DREAM) challenges. We also propose an application of sMOCA for transfer learning where we use pre-trained computational models from a domain where labeled data are abundant and apply them to a different domain with less abundant labeled data.

**Availability and implementation:**

GitHub repository, https://github.com/robert-vogel/moca.

## 1 Introduction

The size and complexity of biological datasets, such as whole genome sequencing, single-cell RNA sequencing, proteomics, and imaging, have expanded substantially in the last decade. Applications of data exploration techniques, as well as predictive analytics, such as machine learning (ML), can be used to uncover novel biological insights or to create predictive models (Ezer and Whitaker 2019).

In fact, several ML-based models such as MammaPrint (Slodkowska and Ross 2009), Decipher (Eric *et al.* 2016) and IDx-DR (Van Der Heijden *et al.* 2018) are already in use in the clinic. However, as it has been documented in the literature (Norel *et al.* 2011, Scialdone *et al.* 2015, Hu and Greene 2018), ML models may not generalize well. This is a serious challenge in critical areas of biomedicine, such as disease diagnostics and prognosis. The poor generalization of ML models (Agarwal *et al.* 2005, Kallus and Zhou 2018) can be ascribed to overfitting and/or biases in the training data. Therefore, extensive and objective evaluation and benchmarking of ML models is critical. Crowd-sourced data competitions, e.g. Dialogue on Reverse Engineering and Methods (DREAM) challenges (Stolovitzky *et al.* 2009, Saez-Rodriguez *et al.* 2016), provide an objective platform to benchmark and validate different algorithms submitted by participants.

Independently trained predictive models can be combined into one aggregate predictive model. The endeavor of combining predictions from multiple algorithms is known as ensemble learning in the ML community (Whalen and Pandey 2013, Kim *et al.* 2021). By not relying on any single model, ensemble learning has the potential to produce more robust algorithms than any of its constituents (Whalen and Pandey 2013). In fact, many of the most popular ML models, such as random forest or boosting algorithms, are ensemble-based predictors.

Crowd-sourced data competitions provide a natural platform for ensemble learning in that dozens of models developed by participating groups can be combined into one ensemble. DREAM challenge organizers have consistently observed in many of their crowd-sourced competitions that even a simple combination strategy such as averaging the submitted predictions has better generalization properties than any of the individual methods (Marbach *et al.* 2010, 2012, Saez-Rodriguez *et al.* 2016). We will call this simple averaging strategy the Wisdom of Crowds (WOC) ensemble throughout the current article.

While simple and effective in many applications, the WOC ensemble is by no means the optimal ensemble strategy. In the DREAM Network Inference Challenge (Marbach *et al.* 2012), e.g. a simple linear ensemble predictor where each algorithm is assigned a weight proportional to its performance was considerably superior to the WOC ensemble. As another example, in the DREAM Digital Mammography challenge (Schaffter *et al.* 2020), a logistic regression-based meta-learner ensemble classifier significantly outperformed each constituent classifier as well as the WOC ensemble. Although these ensemble predictor results are promising, they were typically obtained from problems that had enough labeled data to train an ensemble classifier. However, for many problems in biology and medicine, creating labeled data is very costly, and therefore, we usually have to use unlabeled data or, at the very best, have limited labeled data where training a supervised ensemble classifier is difficult without running into the risk of overfitting.

To bridge this gap, we propose two novel ensemble learning strategies. The first strategy, uMOCA (unsupervised MOCA), works in the context of unsupervised ensemble learning, where there is no sufficient amount of labeled data to train an ensemble classifier. uMOCA takes as input a matrix of predictions of a set of classifiers and, as a first step, estimates the performance of individual classifiers from it. It then forms a linear ensemble classifier in which each individual classifier is assigned a weight proportional to its estimated performance. We show theoretically that the uMOCA algorithm is the optimal linear ensemble under the assumption of class-conditioned independence (i.e. the classifiers make independent predictions given the class labels). Although uMOCA works robustly and accurately in many instances, in applications where the class-conditioned independence assumption is strongly violated, the performance of uMOCA suffers because uMOCA does not accurately estimate base classifier performances, and is no anymore the optimal ensemble. Our second algorithm, sMOCA (supervised MOCA), deals with these situations when a labeled dataset is available. sMOCA uses a labeled dataset to calculate the performance of individual classifiers and forms an optimal linear ensemble by considering the correlation structure of individual algorithms. Compared to supervised ensemble learning methods such as stacking (Whalen and Pandey 2013), where the ensemble classifier is constructed *via* meta-training, sMOCA is less prone to overfitting. This is especially important for problems where only a limited number of samples are available for training the ensemble classifier.

The rest of the article is structured as follows: Firstly, we will introduce the uMOCA ensemble and discuss its performance using simulation data that aligns with our class-conditioned independence assumption. Next, we will introduce the sMOCA algorithm and utilize both the uMOCA and sMOCA in nine DREAM challenges, where the degree of violation of the conditional independence assumption varies. Lastly, we will present a new application of sMOCA in transfer learning and apply it to a skin cancer detection problem where we only have a small amount of labeled data to train individual classifiers.

## 2 Methods

### 2.1 Datasets

The data used in this study were from scientific challenges run by the DREAM and the International Skin Imaging Collaboration (ISIC) archive (Bansal *et al.* 2014, Gutman *et al.* 2016). Detailed information regarding each DREAM challenge can be found at http://dreamchallenges.org/. Specifically, we collected DREAM challenge data from: DREAM 2 BCL6 Transcriptional Target Prediction Challenge (Stolovitzky *et al.* 2009); DREAM 5 Epitope Prediction Challenge, DREAM 5 Network inference using *in silico* and *Escherichia coli* data (Marbach *et al.* 2012); DREAM 8 HPN Network Inference from *in silico* data from Subchallenge 1B (Hill *et al.* 2016); DREAM 8 NIEHS-NCATS-UNC Toxicogenomics Challenge Subchallenge 1 (Eduati *et al.* 2015); and DREAM 9.5 Prostate Cancer Prediction Subchallenge 2 (Seyednasrollah *et al.* 2017). The skin lesion thumbnail image data were collected from MSK1, MSK2, MSK3, and MSK4 datasets from the ISIC-archive (https://www.isic-archive.com/).

### 2.2 Simulations

Simulations were prepared by rank-transforming random samples from two Gaussian distributions given user-specified classifier AUC values and a conditional correlation matrix. We set the mean of the distribution modeling the negative class to zero and the variances representing samples from each class to one. The mean of the positive class distribution was determined from the AUC using the expression AUC = $\Phi [(\mu_{1}-\mu_{0})/\sqrt{\sigma_{1}^{2}+\sigma_{2}^{2}}]$, with Φ representing the standard normal cumulative distribution (Marzban 2004). While the respective distributions have unit variance, the user-specified correlation matrix is used as the covariance matrix. In Fig. 3I, the covariance matrix is the identity matrix, while in Fig. 3K, the correlations were selected at random from the DREAM 9.5 Prostate Challenge Dataset.

### 2.3 Inferring MOCA weights

The MOCA weights determined by the uMOCA algorithm were estimated piecewise. First, the prevalence of the positive class ρ=P(Y=1) and Δ were inferred using the techniques presented in Ahsen *et al.* (2019). Specifically, we implement their iterative procedure for estimating a rank one tensor from the covariance matrix or third central moment tensor using Numpy’s eigenvector decomposition of Hermitian matrices module [numpy.linalg.eigh, (Oliphant 2006)] and the CANDECOMP/PARAFAC decomposition with one leading factor (Kolda and Bader 2009) made readily available in Tensorly (Kossaifi *et al.* 2019), respectively (see Supplemental Note 3 for mathematical details).

### 2.4 Transfer learning for skin lesion classification

Skin lesion images were resized using sci-kit image (van der Walt *et al.* 2014) module “transform.resize.” For each deep learning model from TensorFlow Hub, we trained an *L*_1_ regularized Logistic Regression and Gaussian Naive Bayes classifier using sci-kit learn (Pedregosa *et al.* 2011). For classifier training and testing, we performed 10 unique runs of 5-fold cross-validation for a total of 50 tests. The training set was split into two equal partitions. The first was used to train either the *L*_1_ regularized Logistic Regression or Gaussian Naive Bayes classifiers. In the regularized Logistic Regression, we automated the selection of the regularizer strength by selecting the one which maximized the average AUC of the classifier over 10-fold cross-validation tests. Specifically, we tested 15 regularizer values logarithmically spaced between [0.01, 10]. The second training set was then used to train the sMOCA classifier by estimating the MOCA weights and performing a greedy algorithm for ensemble selection.

The statistical significance of sMOCA outperforming all other methods in terms of AUC, BA, and *F*1 score was assessed by the *t*-test assuming related samples, Wilcoxon rank-sum test, Wilcoxon signed rank test, and binomial test. For all tests, we used the corresponding implementation in the scipy.stats module (Jones *et al.* 2001–), and the 50 estimates of performance from the 10 independent runs of 5-fold cross-validation. To apply the binomial test, we assigned each test comparing sMOCA with an alternative binary value. A one was assigned when the performance of sMOCA was greater than that of the alternative method and zero otherwise. The estimated *P *<* *.001 is used for statistical significance in all measurements.

## 3 Results

**Figure 1.**
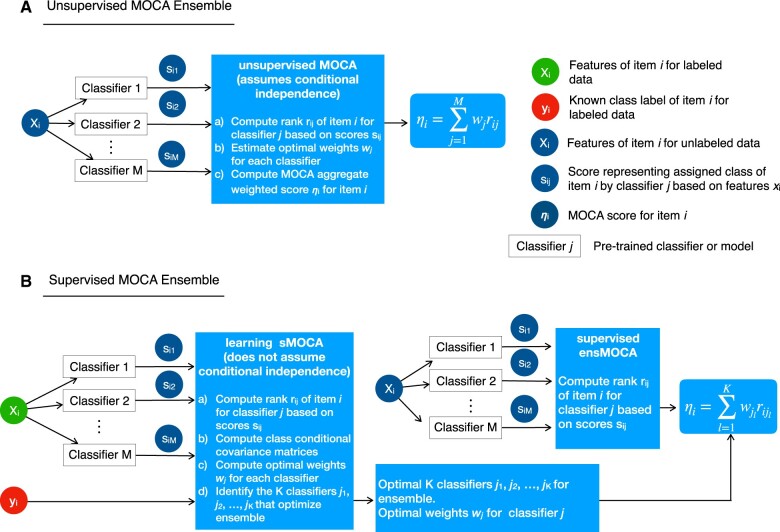
The MOCA strategy. The MOCA strategy is an optimal aggregation of rank-ordered predictions by pre-trained binary classifiers on new, never-before-seen data. It has two versions that can be applied in either the absence or presence of labeled data. (A) When labels are not present, the uMOCA algorithm infers the optimal weights without using any labeled examples. (B) When labels are available, the sMOCA algorithm estimates the optimal weights and greedily selects the optimal combination of base classifiers. sMOCA is noteworthy on account that its training data requirements can be much less than the pre-trained models it is aggregating.

The MOCA classifier is an optimal way of linearly combining class predictions made by a set of binary classifiers, where each classifier is assigned a weight (the MOCA weight) such that the ability of the resulting ensemble classifier to discriminate between the two classes, as measured by the signal-to-noise ratio (SNR) (to be defined below), is maximized. The MOCA ensemble has two variants: (i) uMOCA (Fig. 1A), for applications in which classifiers had been previously trained (supervised or unsupervised), and we do not have labeled data to train an ensemble, and (ii) sMOCA (Fig. 1B) for applications when we have enough labeled data to train the ensemble classifier. In uMOCA (Fig. 1A), we infer the MOCA weights directly from the second and third statistical moments of classifier predictions on a test dataset, which can be calculated without the use of class labels. The uMOCA ensemble is optimal under the assumption that base classifier predictions are conditionally independent given class labels. In sMOCA (Fig. 1B), we use the available sample class labels to calculate the MOCA weights.

### 3.1 The SNR and calculation of MOCA weights

Suppose we are given *M* binary base classifiers and *N* samples out of which *N*_1_ belong to the positive class (*y *=* *1) and $N_{0}=N-N_{1}$ belong to the negative class (*y *=* *0). Each of the *M* base classifiers assigns each sample *i* a score *s_i_* commensurate with its certainty that the sample belongs to the positive (high score) or negative class (low score). For example, in the case of a naive Bayes classifier, this score could be the posterior probability that a given sample belongs to the positive class. In the case of a support vector machine (SVM) classifier, the score could be the distance between the sample and the separating hyperplane in feature space.

Calibration is essential to ensemble learning as different base classifiers may produce scores on different scales (Whalen and Pandey 2013). Take, e.g. the gene network inference problem, where algorithms assign a score indicative of the presence or absence of a direct functional dependence between two genes. These algorithms may produce scores on entirely different scales, such as the Pearson correlation coefficient in WGCNA (Langfelder and Horvath 2008) or mutual information in ARACNE (Algorithm for the Reconstruction of Accurate Cellular Networks) (Margolin *et al.* 2006). The Pearson correlation coefficient takes values in the interval [−1,1], whereas mutual information takes values in [0,∞) (Bishop 2006). Without calibration, the contribution of a base classifier’s score to the ensemble may be biased due to the scale of the produced score and result in inferior ensemble performance. In this work, we use rank transformations of base classifier scores to calibrate classifier predictions. There are two main reasons for using ranks. Firstly, transforming scores to ranks is easy and can be used in an unsupervised way. Secondly, we can calculate important performance metrics such as the area under the receiving operator characteristics curve (AUC) using only the ranking of samples rather than actual scores assigned by an algorithm (Marzban 2004).

**Figure 2.**
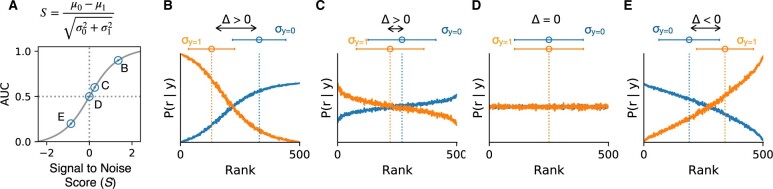
The signal-to-noise score. Simulated rank predictions of 500 samples in which 200 samples (prevalence *ρ *= 0.4) are from the positive class (*y *=* *1). The simulation consists of assuming unit-variance Gaussian class-conditioned score distributions with the differences between the score means for classes 1 and 0 chosen such that the AUC= $\Phi (\frac{\bar{s_{1}}-\bar{s_{0}}}{2})$, where Φ is the standard normal cumulative distribution and $\bar{s_{1}}$ and $\bar{s_{1}}$ are the mean scores for classes 1 and 0, respectively. Estimates of the probability of sample rank given the class label, P(R=r|Y=y), were computed by averaging the true class labels at a given rank over 1000 replicate simulation experiments. (A) The AUC is related to the signal-to-noise score by a sigmoidal function. (B–E) Plots of the conditional distribution for methods with an AUC of (B) 0.9, (C) 0.6, (D) 0.5, and (E) 0.2.

In what follows, we will use the convention that a classifier assigns higher scores to samples it predicts to be more likely to belong to the positive class, and the rank transformation transforms higher scores to lower ranks. For example, the sample that gets the rank 1 is the one for which the classifier produced the largest score and is, therefore, the sample that is most likely to be in the positive class for that classifier. Let *r_ik_* denote the rank of a sample *k* assigned by classifier *i*. To measure the performance of a classifier *i*, we will use the SNR defined as:

(1) $$S_{i}:=\frac{\mu_{i|0}-\mu_{i|1}}{\sqrt{\sigma_{i|1}^{2}+\sigma_{i|0}^{2}}}=\frac{\Delta_{i}}{\sqrt{\sigma_{i|1}^{2}+\sigma_{i|0}^{2}}},$$

where $\mu_{i|y}$ and $\sigma_{i|y}^{2}$ are the class-conditioned mean and variance of ranked classifier predictions, respectively, and $\Delta_{i}:=\mu_{i|0}-\mu_{i|1}$ denotes the difference between the means of class-conditioned rank predictions of classifier *i*. *S_i_* is not without precedent, and in some instances, it is closely related to the AUC. In Marzban (2004), authors showed that the AUC of normally distributed class-conditioned scores is given by $\Phi (\frac{S_{i}}{\sqrt{2}})$, where Φ is the standard normal cumulative distribution and *S_i_* is given in Eq. (1), where the mean *μ* and standard deviation *σ* correspond to the class-conditioned Gaussian scores densities. The SNR is a measure of how different the average ranks for class 0 and class 1 are, re-scaled with a measure of the dispersion of the ranks around their mean for both classes. Figure 2 shows examples of the SNR for different classifiers. Figure 2A shows the relation between the SNR and the AUC for a family of classifiers characterized by class-conditioned rank distribution ranging from good classifiers (Fig. 2B) to worse than random classifiers (Fig. 2E). The AUC is a monotonically increasing sigmoidal function of the classifiers SNR. This relationship can be made more intuitive by inspecting simulation results depicted in Fig. 2B–E. A high-performing classifier, as depicted in Fig. 2B, will have the highest class-conditioned rank probability mass near the extreme ranks [1, *N*], meaning that samples belonging to the positive (negative) classes are more likely to be ranked low (high). In fact, we know that as the number of samples increases, the AUC asymptotically converges to the probability that a classifier assigns positive samples at lower ranks than negative samples. Therefore, a perfect classifier with an AUC of 1 will always rank positive samples below negative ones. This situation corresponds to class 0 and class 1-conditioned probabilities of nonoverlapping support, with a class 1-conditioned rank probability that is equal to $\frac{1}{\rho N}$ for ranks between 1 and *ρN* and 0 otherwise, and a class 0-conditioned rank probability that is equal to $\frac{1}{(1-\rho )N}$ for ranks between ρN+1 and *N* and 0 otherwise, where *ρ* denotes the proportion of class 1 samples. In this limiting case, the SNR takes its maximum possible value and can be explicitly computed to be

(2) $$\begin{array}{c} SNR(\rho ,N;\mathrm{AUC}=1)=\sqrt{3}/\sqrt{{\left(1-\rho \right)}^{2}+\rho^{2}-2/N^{2}}\\ \leq \sqrt{6}/\sqrt{1-4/N^{2}}. \end{array}$$

The last inequality corresponds to the case ρ=1/2. For ρ=0.4 and *N *=* *200, the maximum SNR for which AUC = 1 is 2.4, as can be seen in Fig. 2A. An uninformative classifier, on the other hand, has a uniform conditional probability for all ranks, which implies that the classifier cannot distinguish positive samples from negative ones (Fig. 2D), and therefore, in this case, the SNR = 0. We would also like to note that a negative SNR is indicative of a base classifier that either incorrectly adopts our convention and assigns samples from the positive class high ranks or has systematic errors that result in the classification of negative samples as positive and vice versa (Fig. 2A and E).

Next, we compute the SNR for an ensemble of *M* classifiers. Let us assume that classifier *i* assigns sample *k* the rank *r_ik_*. If classifier *i* is given a weight *w_i_*, we define the ensemble score $s_{k}^{w}$ for the set of weights w as:

(3) $$s_{k}^{w}=\sum_{i=1}^{M}w_{i}r_{ik}.$$

The SNR for the ensemble, denoted by $S_{\mathrm{ens}}^{w}$, is defined as the difference between the 0 and class 1-conditioned expected values of the ensemble score, normalized by the square root of the sums of class 0 and class 1-conditioned variances of the ensemble score. This is a natural generalization of the definition of the SNR for one classifier given in Eq. (1). We show in Supplementary Note 1 that the SNR of the ensemble can be written as:

(4) $$S_{\mathrm{ens}}^{w}=\frac{w^{T}\Delta }{\sqrt{w^{T}Cw}},$$

where $\Delta ,w\in R^{M\times 1}$ and $C\in R^{M\times M}$. In Eq. (4), Δ and **w** are vectors whose *i*th elements represent the difference in the class-conditioned expected values ($\Delta_{i}:=\mu_{i|0}-\mu_{i|1}$) and ensemble weight *w_i_* assigned to the *i*th base classifier, respectively. The matrix $C:=C_{0}+C_{1}$ is the sum of the class-conditioned covariance matrices (*C_y_*) of the classifiers (see Supplementary Note 1 for details).

Equation (4) is of primary interest, as it defines the ensemble SNR in terms of the classifier weights. It would be desirable to choose weights that maximize the SNR of the ensemble. We will call such weights the MOCA weights and denote them by $w^{\text{MOCA}}$. We show in Supplementary Note 2 that, when normalized to have a norm equal to 1, MOCA weights are given by

(5) $$w^{\text{MOCA}}=\frac{C^{-1}\Delta }{\left|\left|C^{-1}\Delta \right|\right|}.$$

Note that the MOCA weights given in (5) are equivalent to weights assigned to each feature in Fisher’s LDA (Fisher 1936, Xanthopoulos *et al.* 2013). The biggest difference is in MOCA we work in the rank-transformed predictions.

Using this expression in Eq. (4), we find that the MOCA ensemble SNR can be expressed as:

(6) $$S_{\mathrm{ens}}^{w^{\text{MOCA}}}=\left|\left|C^{-1/2}\Delta \right|\right|,$$

where $C^{-1/2}$ is the square root of $C^{-1}$, which exists given that **C** is symmetric and positive definite. Equation (5) shows that the MOCA weight of classifier *i* is a linear combination of the $\Delta_{j}$’s of all classifiers weighted by elements of the inverse of matrix **C**. It is instructive to consider the case for which the off-diagonal elements of **C** are 0, which occurs when the base classifiers are conditionally independent. In this case **C** is diagonal, with diagonal elements $C_{ii}=\sigma_{i|1}^{2}+\sigma_{i|0}^{2}$ and $w_{i}^{\text{MOCA}}\propto \Delta_{i}/(\sigma_{i|1}^{2}+\sigma_{i|0}^{2})$. In this case, the ensemble SNR becomes

(7) $$S_{\mathrm{ens}}^{w^{\text{MOCA}}}=\sqrt{\sum_{i=1}^{M}\frac{\Delta_{i}^{2}}{\sigma_{i|1}^{2}+\sigma_{i|0}^{2}}}.$$

Note that when *M *=* *1, expression (7) reduces to Eq. (1), the SNR for one classifier, which has an upper bound given by Eq. (2). However, as *M* increases, the MOCA ensemble SNR grows as $\sqrt{M}$ provided the SNRs of the base classifiers in the ensemble are bigger than 0, even if they are weak classifiers. This suggests that in the case of class-conditioned independent classifiers, the MOCA ensemble approaches the perfect classifier as the number of classifiers *M* increases.

However, when the off-diagonal elements of the conditional covariance matrices are not zero, the denominator of each of the Δ_*i*_ terms in Eq. (6) increases, and in effect, down-weights the individual contributions of highly correlated methods to the ensemble as they do not carry independent information. In consequence, MOCA weights automatically balance the performance of base classifiers and the diversity of their predictions. These two properties together, performance and classifier diversity, allow the ensemble performance to exceed that of its constituents (Whalen and Pandey 2013). In the next section, we show how to calculate the difference in conditional means Δ_*i*_ and the covariance matrices $C_{0}$ and $C_{1}$ required to compute the MOCA weights using Eq. (5) in the case of unlabeled data.

### 3.2 Unsupervised estimation of MOCA weights

We first apply the MOCA ensemble in an unsupervised setting. In the absence of sample class labels, we are unable to calculate the class-conditioned means and covariance matrices of sample rank predictions required to compute the MOCA weights. To make progress, we will add the hypothesis that the classifiers in our ensemble are conditionally independent given the class, also assumed in previous work (Parisi *et al.* 2014, Jaffe *et al.* 2015, Ahsen *et al.* 2019). Under this hypothesis, matrix **C** is diagonal, and we only need to estimate the conditional mean and variance for each classifier in order to construct the MOCA ensemble.

Even in the absence of labeled data, it is remarkable that it is possible to estimate the MOCA weights $w^{\text{MOCA}}$ under the assumption of class-conditioned independent classifier predictions. We showed in the previous section that for conditionally independent classifiers, the MOCA weights are given by

(8) $$w_{i}^{\text{MOCA}}=\beta \frac{\Delta_{i}}{\sigma_{i|0}^{2}+\sigma_{i|1}^{2}},$$

where *β* is a constant so that the vector $w^{\text{MOCA}}$ has unit norm: $\beta ={\left[\sum_{i=1}^{M}\Delta_{i}^{2}/(\sigma_{i|1}^{2}+\sigma_{i|0}^{2})\right]}^{-1/2}$. In Ahsen *et al.* (2019), the authors show how to estimate Δ_*i*_ from unlabeled data, which we use in estimating MOCA weight under the assumption of conditional independence of classifier predictions. The conditional independence assumption ensures that the covariance ensures that the only contributing factor to covariance is the discriminating ability of classifiers [for more info, see Ahsen *et al.* (2019)]. The final piece needed to estimate the MOCA weights, therefore, is to determine $\sigma_{i|0}^{2}+\sigma_{i|1}^{2}$, the sum of class-conditioned variances of each base classifier *j*. In this work, we provide a novel way to estimate the sum of the conditional variances from the second- and third-order moments of the unconditioned rank predictions (see Supplementary Note 3 for derivation and details).

**Figure 3.**
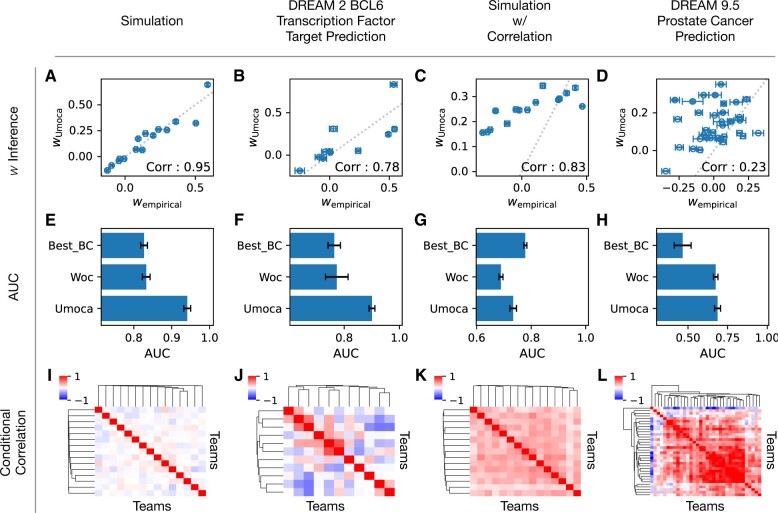
The unsupervised MOCA algorithm. MOCA was applied to (A, E, I) simulation data where base classifiers predictions are conditionally independent, (B, F, J) predictions by teams participating in the DREAM2 BCL6 Transcription factor target prediction challenge, (C, G, K) simulation data where base classifier predictions are conditionally dependent, and (D, H, L) predictions by teams participating in the DREAM 9.5 Prostate Cancer Prediction Challenge. For each dataset, we demonstrate MOCA’s ability to infer MOCA weight, *w_i_*, i=1,2,…,M, measure the AUC in relation to the wisdom of crowd ensemble (WOC), and the best individual base classifier (Best_BC), and measure the empirical conditional correlation matrix *C*. The error bars represent SEM computed from 5-fold cross-validation.

We first tested the uMOCA strategy by simulating predictions of conditionally independent base classifiers (Fig. 3I). In this simulated setting, uMOCA was able to accurately infer the MOCA weights, achieving a correlation between the real MOCA weights (calculated using actual class labels) and the inferred MOCA weights of 0.99 (Fig. 3A). Using the MOCA weights in the ensemble score given in Eq. (3), we can assign a score to each sample to compute the AUC of the ensemble. A good point of comparison for uMOCA is a simple ensembling methodology, which we will call the WOC ensemble (Marbach *et al.* 2012), consisting of making all weights in the ensemble be equal to 1, that is $w_{i}^{\mathrm{WOC}}=1$. We next compare uMOCA and WOC ensemble classifiers using the AUC as the performance metric. In Fig. 3E, we see that the uMOCA classifier outperforms the WOC ensemble and the best-performing individual classifier. The inferior performance of the WOC ensemble can be attributed to the fact that good and bad predictions are equally weighted. The MOCA ensemble gives more weight to the better classifiers and less weight to the worse classifiers, making the predictions more accurate. Next, we applied uMOCA to the problem of gene network inference. Here, we collected predictions from teams participating in the DREAM2 BCL6 Transcription Factor Prediction Challenge (Stolovitzky *et al.* 2009). In this challenge, participating teams were asked to predict which of the 200 genes are transcriptional targets of the transcription factor BCL6 and which ones are decoys. Gold standard labels were experimentally determined for these genes and used by the challenge organizers to benchmark participants’ algorithms. When applying uMOCA to the rank-transformed predictions, we found that the inferred MOCA weight and the true weights computed using the gold standard labels had a correlation of 0.81 (Fig. 3B). The inferred MOCA weights were then used to assign a MOCA ensemble score to each sample. The AUC of the MOCA ensemble outperformed the best base prediction as well as the WOC ensemble (Fig. 3F). We attribute the good performance of uMOCA to the fact that our conditional independence assumption is not significantly violated in these rank predictions. This can be qualitatively assessed by inspecting the off-diagonal entries of the normalized conditional covariance matrix $C=C_{0}+C_{1}$ shown as heat maps in the bottom row of Fig. 3. In Fig. 3I, the off-diagonal entries are close to zero, as is expected from conditionally independent simulation data. In comparison, Fig. 3J shows small deviations from zero-correlation for the actual challenge data. We conclude that uMOCA is robust to moderate violations of the conditional independence assumption. We next investigated the performance of uMOCA in cases for which our assumption of conditional independence is strongly violated. First, we use simulation data with conditionally dependent predictions (Fig. 3K) and see that the accuracy of inferring the MOCA weights (Fig. 3C) is less than its conditionally independent counterpart (Fig. 3A). In effect, the AUC of the uMOCA classifier is less than the best individual method (Fig. 3G). We then applied uMOCA to the DREAM Prostate Cancer Prediction challenge. In the DREAM Prostate Cancer Prediction challenge, participants were asked to predict outcomes survival for prostate cancer patients based on patients’ clinical variables (Guinney *et al.* 2017). The participants were given clinical covariates from four clinical trials. In applying uMOCA, we see that the MOCA weights are inferred with a correlation of 0.23 (Fig. 3D). Here, the performance of the uMOCA classifier is not statistically significantly different than either WOC or the best individual classifier (Fig. 3H). We attribute this poor performance to the strong conditional covariance between base classifier predictions (Fig. 3L). There are two main reasons for the poor performance of uMOCA ensemble in this case. First, the elements that go into the computation of the MOCA weights (Δ, *ρ*, and $\sigma_{i|1}^{2}+\sigma_{i|0}^{2})$ are not estimated accurately. Second in forming the functional form of the uMOCA weights is the result of optimizing the ensemble SNR under the assumption of conditional independence. Once this assumption is violated, the uMOCA weights are no longer optimal, and we need to account for the correlation between classifiers. In the next section, we show how we can overcome these shortcomings when sample labels are available.

Various unsupervised clustering methods exist in the literature, such as GMM (Gaussian Mixture Models) (Yang *et al.* 2012) and Naive Bayes Clustering (Gamez *et al.* 2006). Similar to uMOCA, the naive Bayes approach assumes class conditional independence of predictions, and similar to GMM, it uses Gaussian priors. To see how uMOCA compares to those clustering methods, we compared uMOCA to GMM (see Supplementary Note 5). Our results show that uMOCA performs better than GMM when the conditional independence assumption is not violated. When the conditional independence assumption is violated, uMOCA seems to have to clear advantage over GMM. This advantage of uMOCA could be due to several reasons, as we explain below. For unsupervised clustering methods such as GMM, the decision boundary between modes could be more flexible than the uMOCA. Furthermore, the data does not need to be exactly Gaussian for a GMM to perform well, even better than uMOCA. But this cannot be known a priori. What uMOCA provides is a more principled, optimal method under the appropriate conditions.

### 3.3 Supervised MOCA

We next applied MOCA in settings in which labeled samples to train the ensemble classifier exist. In such a setting, MOCA weights can be estimated directly from the empirical class-conditioned means and covariance matrices. Therefore, the conditional independence assumption is not required. We call this use of MOCA supervised MOCA, or sMOCA for short. In cases where we have only a limited amount of labeled samples, applying an optimal linear combination such as MOCA is less prone to overfitting. This is because we use the labels only to compute the first and second-class-conditioned moments of the rank predictions rather than training a supervised ensemble algorithm, which typically requires splitting the training data and using cross-validation schemes.

We first run the sMOCA algorithm in the DREAM BCL6 Challenge and DREAM Prostate Cancer Prediction Challenge data discussed in the previous section. In Table 1, we see that sMOCA achieves an AUC = 0.915 ± 0.018 while uMOCA has an AUC = 0.901 ± 0.023. In this challenge, the performance improvement of sMOCA over uMOCA is not very dramatic as the conditional independence assumption necessary for the use of uMOCA is only slightly violated, and therefore, the estimates obtained using uMOCA are reasonably accurate. However, the conditional independence assumption is strongly violated for the DREAM Prostate Cancer Prediction Challenge. This is reflected in the performance comparison between sMOCA and uMOCA. sMOCA achieves an AUC = 0.726 which exceeds the AUC of uMOCA and WOC by nearly 0.04 and more than 0.05, respectively.

**Table 1.** Comparison analysis of uMOCA and sMOCA.^a^

Challenge	Unsupervised	Supervised	WOC	Best individual
	MOCA	MOCA		Method
D2: Bcl6 transcription factor target prediction	0.901 ± 0.023	0.915 ± 0.018	0.804 ± 0.029	0.848 ± 0.032
D5: Epitope prediction	0.874 ± 0.002	0.900 ± 0.002	0.872 ± 0.002	0.893 ± 0.003
D5: In silico network inference	0.781 ± 0.005	0.857 ± 0.002	0.810 ±	0.816 ± 0.004
D5: E coli network inference	0.645 ± 0.007	0.722 ± 0.007	0.681 ± 0.009	0.671 ± 0.008
D7: NCI drug combination: synergy	0.715 ± 0.090	0.853 ± 0.046	0.497 ± 0.063	0.860 ± 0.014
D7: NCI drug combination: antagonism	0.510 ± 0.107	0.803 ± 0.056	0.555 ± 0.079	0.648 ± 0.027
D8: HPN Network Inference SC1B *in silico* predictions	0.538 ± 0.058	0.804 ± 0.019	0.582 ± 0.031	0.720 ± 0.026
D8: NIEHS-NCATS-UNC Toxicogenomics	0.904 ± 0.002	0.905 ± 0.002	0.903 ± 0.002	0.904 ± 0.002
D9.5: Prostate cancer prediction	0.687 ± 0.029	0.726 ± 0.011	0.672 ± 0.032	0.656 ± 0.020

a Each entry represents the mean AUC ± SEM from 5-fold cross-validation using data from unique scientific challenges.

We performed a more extensive comparative analysis by measuring the performance of uMOCA, sMOCA, WOC, and the best individual method in seven additional DREAM challenges. The complete set of challenges represents diverse problems in computational biomedicine, including drug combination synergy and antagonism prediction, network inference, toxicogenomics, and epitope prediction. In all but a single example, we observed that sMOCA is the top-performing method in terms of AUC (Table 1). In the DREAM NCI drug combination antagonism prediction challenge and the HPN Network Inference Challenge, sMOCA outperforms each method by an AUC of greater than 0.15 and 0.08, respectively (Table 1).

### 3.4 MOCA for transfer learning

So far, we have shown how to use MOCA in cases that aim to combine a set of trained base classifiers. Transfer learning is a field of ML where models pre-trained in one domain are repurposed in similar domains (Pan and Yang 2010). Transfer learning is increasingly used in various biomedical problems, including cancer detection from medical images such as mammograms. For example, in the recent DREAM Digital Mammography challenge (Schaffter *et al.* 2020), participants used deep learning methods pre-trained on computer vision datasets such as ImageNet (Deng *et al.* 2009) and repurposed those models to predict breast cancer from mammograms. Given that there are various models for re-purposing, it is not known which model generalizes better to the new domain. Therefore, instead of relying on one pre-trained algorithm, we can use MOCA and combine multiple algorithms.

To test the MOCA strategy for transfer learning we collected five pre-trained models and 2750 skin lesion thumbnail images. The pre-trained models, obtained from Google’s TensorFlow (Abadi *et al.* 2016) and TensorFlow Hub, are Inception v3 (Szegedy *et al.* 2016), PNASNet-Large (Liu *et al.* 2018, Zoph *et al.* 2017), ResNet-v2 (He *et al.* 2016), and MobileNet-v2 (Sandler *et al.* 2018). The dataset has 2750 skin lesion thumbnail images collected from the publicly available ISIC-archive (Gutman *et al.* 2016). Although 2750 images may seem like a good-size dataset, when combined with its counterparts in image processing with millions of images, the 2750 skin lesion images represent a very modest number of labeled examples to effectively fine-tune each, or any, of the deep learning models.

**Figure 4.**
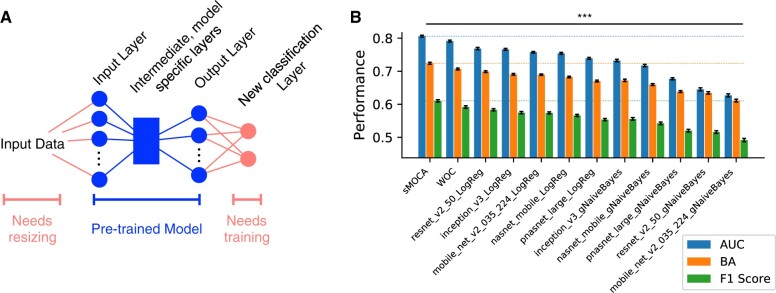
Transfer learning with sMOCA was applied to automated melanoma classification using 2750 images from the ISIC-archive and five deep learning models from TensorFlow Hub: (i) inception_v3, (ii) mobile_net_v2_035_224, (iii) resetnet_v2, (iv) pnasnet_large, and (v) nasnet_mobile. (A) Each deep learning model was pre-trained on the ImageNet 2012 (ILSVRC-2012-CLS) dataset. To apply to images for melanoma prediction, we resized images to match the input layer of the respective network and then used the output layer values for each image as a feature vector for binary classification by either *L*_1_ regularized Logistic Regression or Gaussian Naive Bayes. We then assessed the performance of each deep learning model paired with a binary classifier for a total of 10 independent methods by 10 independent rounds of 5-fold cross-validation. In each fold, we split the training data into two groups, the first for training the classification layer and the second for training sMOCA. (B) The bar chart shows the average performance as measured by AUC, BA, and *F*1 score ± SEM for sMOCA, WOC, and the independent methods. sMOCA outperformed, with respect to each performance measure, all other methods with *P *<* *.001.

The pre-trained models could not be blindly applied to the new dataset. Indeed, many of the models required input images of unique size, and their output layers produced a unique number of features. As such, we used standard image interpolation to resize the images and appended the respective output layer with a binary classification layer (Fig. 4A). For each of the five models, we trained two distinct classification layers, making a total of 10 unique base classifiers. The classifiers we chose were *L*_1_ regularized Logistic Regression and Gaussian Naive Bayes, as they represent two distinct training strategies of the same sigmoidal function, whose relative performances were not *a priori* known (Ng and Jordan 2002). Indeed, the application of these modest additions to each deep learning model for the classification of 2750 images was completed with off-the-shelf tools and using a laptop CPU with modest memory in less than a day.

Figure 4B presents the performance of each base classifier, the WOC and sMOCA in terms of AUC, the balanced accuracy (BA) [0.5 (TPR + TNR)] and *F*1 score (harmonic mean of the precision and recall). Here, we see that sMOCA significantly outperforms all other methods in terms of AUC. Moreover, sMOCA outperforms all other strategies in terms of the BA and *F*1 scores. This is of interest because BA and *F*1 score evaluate the ability of a classifier to correctly infer sample class labels. An important ability for computer-aided diagnosis is the classification of an ailment as opposed to the assignment of a continuous score. Together we see that sMOCA for transfer learning outperforms WOC and its constituents in generating sample scores (AUC) and in inference of sample class labels (BA and *F*1 score).

## 4 Conclusion

Many common biomedical tasks, such as cancer diagnostics, differential expression calling, somatic mutation calling, and gene network inference, can be viewed as binary classification problems. For example, in a differential expression analysis, we are trying to predict genes whose expression significantly differs between two conditions (e.g. treatment versus control). Most differential expression callers output *P*-values, which can be used to rank genes from the most likely to be differentially expressed between the two conditions of interest to the least likely. Many tools, such as limma (Smyth 2005) and DESeq (Anders and Huber 2012), are proposed for the differential expression analysis. Different algorithms have distinct assumptions on the data analyzed, which in a real experimental setup is not known *a priori* nor can it be estimated given the fact that most experiments consist of only a few biological replicates. Therefore, given the unlabeled nature of this problem, it is not known which algorithm will work the best in a given context. In such problems, choosing an ensemble of different algorithms will perform more robustly than any individual algorithm.

In this article, we first introduced the uMOCA algorithm which deals with unsupervised problems such as the differential expression calling. uMOCA estimates the performance of each classifier in terms of AUC and produces an optimal ensemble classifier by assigning each classifier a weight proportional to its estimated performance which is exact when the individual classifiers produce conditionally independent predictions. We then empirically showed in a simulated example and in a DREAM crowdsourcing challenge (BCL6 DREAM Challenge) that uMOCA performs significantly better than any single algorithm and the WOC strategy. Unlike many traditional supervised learning problems where models are trained using labeled data, in this challenge, the participants created models based on first-order principles as well as a diverse range of external datasets. This resulted in a diversity of individual models that did not violate our conditional independence assumption significantly, leading to the superior performance of uMOCA. We also compared uMOCA to unsupervised learning methods such as GMM. Our empirical results show that uMOCA performance is better than GMM in cases where classifiers are conditionally independent, and there is no significant difference between GMM and uMOCA when the conditional independence assumption is violated.

Although uMOCA performs robustly in different tasks, it is no longer the optimal ensemble classifier when the classifier predictions are strongly conditionally dependent. The conditional independence assumption might be violated if the base classifiers are similar (typical for supervised learning problems) and/or trained on the same dataset. To deal with such cases, we proposed the sMOCA algorithm, which uses class labels for estimating the MOCA weights and performs optimal ensemble selection. This is especially useful in supervised problems where there are only a handful of samples to create the ensemble classifier. In such cases training a meta-classifier might lead to overfitting, whereas a simple theoretically backed strategy as sMOCA is more robust. Using nine crowdsourcing challenges, we showed that in eight challenges, sMOCA significantly outperformed uMOCA, the best individual method, as well as the WOC strategy. Finally, we proposed a novel use of sMOCA in a transfer learning problem where we use deep learning methods pre-trained on the large annotated dataset ImageNet, to predict skin cancer where the amount of labeled dataset was less than 1% of the ImageNet dataset. Our analysis of this dataset showed that there is value in using ensemble strategies in transfer learning problems. We can significantly improve any individual model by using the simple WOC strategy. The application of sMOCA to this dataset further improved the WOC performance.

Our results show that applying ensemble strategies improves the robustness of predictive methods in various supervised and unsupervised biomedical problems. In inherently unlabeled problems such as gene network inference and differential expression calling, uMOCA can greatly enhance the prediction performance by optimally combining predictions from different models. If there is a labeled dataset to create an ensemble classifier, the sMOCA algorithm should be preferred as it can handle dependence between classifiers. sMOCA is especially useful in cases where there is only a limited number of samples, and traditional supervised algorithms are prone to overfitting.

## Supplementary Material

vbae093_Supplementary_Data

## Data Availability

The GitHub repository, located at https://github.com/robert-vogel/moca, hosts all the necessary data in the article and code for running the MOCA package.

## References

[vbae093-B1] Abadi M , Barham P, Chen J et al Tensorflow: a system for large-scale machine learning. In: *12th USENIX Symposium on Operating Systems Design and Implementation (OSDI 16)*, Savannah, GA, USA, 2016, 265–83.

[vbae093-B2] Agarwal S , Graepel T, Herbrich R et al Generalization bounds for the area under the roc curve. J Mach Learn Res 2005;6:393–425.

[vbae093-B3] Ahsen ME , Vogel RM, Stolovitzky GA. Unsupervised evaluation and weighted aggregation of ranked classification predictions. J Mach Learn Res 2019;20:1–40.

[vbae093-B4] Anders S , Huber W. Differential Expression of RNA-seq Data at the Gene Level – The DESeq Package. Heidelberg, Germany: European Molecular Biology Laboratory (EMBL), 2012.

[vbae093-B5] Bansal M , Yang J, Karan C et al A community computational challenge to predict the activity of pairs of compounds. Nat Biotechnol 2014;32:1213–22. 25419740 10.1038/nbt.3052 PMC4399794

[vbae093-B6] Bishop CM. Pattern Recognition and Machine Learning. New York: Springer, 2006.

[vbae093-B7] Deng J , Dong W, Socher R et al Imagenet: a large-scale hierarchical image database. In: *2009 IEEE Conference on Computer Vision and Pattern Recognition*, Miami, FL, USA, 2009, 248–55.

[vbae093-B8] Eduati F , Mangravite LM, Wang T et al Prediction of human population responses to toxic compounds by a collaborative competition. Nat Biotechnol 2015;33:933–40. 26258538 10.1038/nbt.3299 PMC4568441

[vbae093-B9] Eric A , Klein Z, Haddad K et al Decipher genomic classifier measured on prostate biopsy predicts metastasis risk. Urology 2016;90:148–52. 26809071 10.1016/j.urology.2016.01.012

[vbae093-B10] Ezer D , Whitaker K. Data science for the scientific life cycle. Elife 2019;8:e43979. 30839275 10.7554/eLife.43979 PMC6402833

[vbae093-B11] Fisher RA. The use of multiple measurements in taxonomic problems. Ann Eugen 1936;7:179–88.

[vbae093-B12] Gamez JA , Rumí R, Salmeron A. Unsupervised naive bayes for data clustering with mixtures of truncated exponentials. In: Probabilistic Graphical Models. 2006, 123–30.

[vbae093-B13] Guinney J , Wang T, Laajala TD et al Prediction of overall survival for patients with metastatic castration-resistant prostate cancer: development of a prognostic model through a crowdsourced challenge with open clinical trial data. Lancet Oncol 2017;18:132–42. 27864015 10.1016/S1470-2045(16)30560-5 PMC5217180

[vbae093-B14] Gutman D , Codella NCF, Celebi E et al Skin lesion analysis toward melanoma detection: a challenge at the International Symposium on Biomedical Imaging (ISBI) 2016, hosted by the International Skin Imaging Collaboration (ISIC). In: *2018 IEEE 15th International Symposium on Biomedical Imaging (ISBI 2018)*, Washington, DC, USA, 2018, 168–72. doi: 10.1109/ISBI.2018.8363547.

[vbae093-B15] He K , Zhang X, Ren S et al Identity mappings in deep residual networks. In: *European Conference on Computer Vision*. Amsterdam, The Netherlands: Springer, 2016, 630–45.

[vbae093-B16] Hill SM , Heiser LM, Cokelaer T et al Inferring causal molecular networks: empirical assessment through a community-based effort. Nat Methods 2016;13:310–8. 26901648 10.1038/nmeth.3773 PMC4854847

[vbae093-B17] Hu Q , Greene CS. Parameter tuning is a key part of dimensionality reduction via deep variational autoencoders for single cell RNA transcriptomics. bioRxiv, 10.1101/385534, 2018, preprint: not peer reviewed. PMC6417816 30963075

[vbae093-B18] Jaffe A , Nadler B, Kluger Y. Estimating the accuracies of multiple classifiers without labeled data. In: Artificial Intelligence and Statistics, PMLR, San Diego, California, USA, 2015, 407–15.

[vbae093-B19] Jones E , Oliphant T, Peterson P et al SciPy: open source scientific tools for Python, 2001–.http://www.scipy.org/. 2001.

[vbae093-B20] Kallus N , Zhou A. Residual unfairness in fair machine learning from prejudiced data. In: *Proceedings of the 35th International Conference on Machine Learning*, PMLR 80, 2018, 2439–48.

[vbae093-B21] Kim S-C , Arun AS, Ahsen ME et al The fermi–dirac distribution provides a calibrated probabilistic output for binary classifiers. Proc Natl Acad Sci USA 2021;118:e2100761118. 34413191 10.1073/pnas.2100761118 PMC8403970

[vbae093-B22] Kolda TG , Bader BW. Tensor decompositions and applications. SIAM Rev 2009;51:455–500.

[vbae093-B23] Kossaifi J , Panagakis Y, Anandkumar A et al Tensorly: tensor learning in python. J Mach Learn Res 2019;20:1–6.

[vbae093-B24] Langfelder P , Horvath S. WGCNA: an R package for weighted correlation network analysis. BMC Bioinformatics 2008;9:559. 19114008 10.1186/1471-2105-9-559 PMC2631488

[vbae093-B25] Liu C , Zoph B, Shlens J et al Progressive neural architecture search. In: *Proceedings of the European Conference on Computer Vision (ECCV)*, 2018, 19–34.

[vbae093-B26] Marbach D , Costello JC, Küffner R et al Wisdom of crowds for robust gene network inference. Nat Methods 2012;9:796–804. 22796662 10.1038/nmeth.2016 PMC3512113

[vbae093-B27] Marbach D , Prill RJ, Schaffter T et al Revealing strengths and weaknesses of methods for gene network inference. Proc Natl Acad Sci USA 2010;107:6286–91. 20308593 10.1073/pnas.0913357107 PMC2851985

[vbae093-B28] Margolin AA , Nemenman I, Basso K et al ARACNE: an algorithm for the reconstruction of gene regulatory networks in a mammalian cellular context. BMC Bioinformatics 2006;7:S7. 10.1186/1471-2105-7-S1-S7 PMC1810318 16723010

[vbae093-B29] Marzban C. The roc curve and the area under it as performance measures. Weather and Forecast 2004;19:1106–14.

[vbae093-B30] Ng AY , Jordan MI. On discriminative vs. generative classifiers: a comparison of logistic regression and naive bayes. In: *Advances in Neural Information Processing Systems*. 2002, 841–8.

[vbae093-B31] Norel R , Rice JJ, Stolovitzky G. The self-assessment trap: can we all be better than average? Mol Syst Biol 2011;7:537. 21988833 10.1038/msb.2011.70 PMC3261704

[vbae093-B32] Oliphant TE. A Guide to NumPy, Birmingham, UK: Trelgol Publishing, 2006.

[vbae093-B33] Pan SJ , Yang Q. A survey on transfer learning. IEEE Trans Knowl Data Eng 2010;22:1345–59.

[vbae093-B34] Parisi F , Strino F, Nadler B et al Ranking and combining multiple predictors without labeled data. Proceedings of the National Academy of Sciences 2014;111:1253–8. 10.1073/pnas.1219097111 PMC3910607 24474744

[vbae093-B35] Pedregosa F , Varoquaux G, Gramfort A et al Scikit-learn: machine learning in python. J Mach Learn Res 2011;12:2825–30.

[vbae093-B36] Saez-Rodriguez J , Costello JC, Friend SH et al Crowdsourcing biomedical research: leveraging communities as innovation engines. Nat Rev Genet 2016;17:470–86. 27418159 10.1038/nrg.2016.69 PMC5918684

[vbae093-B37] Sandler M , Howard A, Zhu M et al Mobilenetv2: inverted residuals and linear bottlenecks. In: *2018 IEEE/CVF Conference on Computer Vision and Pattern Recognition*, Salt Lake City, UT, USA, 2018, 4510–20.

[vbae093-B38] Schaffter T , Buist DSM, Lee CI et al Evaluation of combined artificial intelligence and radiologist assessment to interpret screening mammograms. JAMA Network Open 2020;3:e200265. 32119094 10.1001/jamanetworkopen.2020.0265 PMC7052735

[vbae093-B39] Scialdone A , Natarajan KN, Saraiva LR et al Computational assignment of cell-cycle stage from single-cell transcriptome data. Methods 2015;85:54–61. 26142758 10.1016/j.ymeth.2015.06.021

[vbae093-B40] Seyednasrollah F , Koestler DC, Wang T et al A dream challenge to build prediction models for short-term discontinuation of docetaxel in metastatic castration-resistant prostate cancer. JCO Clin Cancer Inform 2017;1:1–15. 10.1200/CCI.17.00018 PMC6874023 30657384

[vbae093-B41] Slodkowska EA , Ross JS. Mammaprint™ 70-gene signature: another milestone in personalized medical care for breast cancer patients. Expert Rev Mol Diagn 2009;9:417–22. 19580427 10.1586/erm.09.32

[vbae093-B42] Smyth GK. Limma: linear models for microarray data. In: Bioinformatics and Computational Biology Solutions using R and Bioconductor. New York: Springer, 2005, 397–420.

[vbae093-B43] Stolovitzky G , Prill RJ, Califano A. Lessons from the dream2 challenges: a community effort to assess biological network inference. Ann N Y Acad Sci 2009;1158:159–95. 19348640 10.1111/j.1749-6632.2009.04497.x

[vbae093-B44] Szegedy C , Vanhoucke V, Ioffe S et al Rethinking the inception architecture for computer vision. In: *2016 IEEE Conference on Computer Vision and Pattern Recognition (CVPR)*, Las Vegas, NV, USA, 2016, 2818–26.

[vbae093-B45] Van Der Heijden AA , Abramoff MD, Verbraak F et al Validation of automated screening for referable diabetic retinopathy with the IDx-Dr device in the Hoorn diabetes care system. Acta Ophthalmol 2018;96:63–8. 29178249 10.1111/aos.13613 PMC5814834

[vbae093-B46] van der Walt S , Schönberger JL, Nunez-Iglesias J et al scikit-image: image processing in Python. PeerJ 2014;2:e453. 25024921 10.7717/peerj.453 PMC4081273

[vbae093-B47] Whalen S , Pandey G. A comparative analysis of ensemble classifiers: case studies in genomics. In: *2013 IEEE 13th International Conference on Data Mining*, Dallas, TX, USA, 2013, 807–16.

[vbae093-B48] Xanthopoulos P , Pardalos PM, Trafalis TB et al Linear discriminant analysis. In: Robust Data Mining. New York: Springer, 2013, 27–33.

[vbae093-B49] Yang M-S , Lai C-Y, Lin C-Y. A robust EM clustering algorithm for gaussian mixture models. Pattern Recognit 2012;45:3950–61.

[vbae093-B50] Zoph B , Vasudevan V, Shlens J et al Learning transferable architectures for scalable image recognition. In: *2018 IEEE/CVF Conference on Computer Vision and Pattern Recognition*, Salt Lake City, UT, USA, 2018, 8697–710.

