# Supplementary material for: Optimal linear ensemble of binary classifiers
Source: Bioinform Adv. 2024 Jun 25;4(1):vbae093. doi: 10.1093/bioadv/vbae093 (PMC11249386; doi:10.1093/bioadv/vbae093)
Supplement: vbae093_Supplementary_Data [file vbae093_supplementary_data.pdf]

Supplementary Note 1 Ensemble Signal to Noise Score

Assume that we have M classifiers $g_i|_{i=1}^M$ that assign each sample, $k \in \{1, \cdots, N\}$, a score commensurate to its probability of belonging to the positive class. These scores can be used to rank the samples, with the most probable belonging to the positive class (low rank) to the least probable (high rank). Similar to [1], let r_{ik} denote the rank assigned to sample k by classifier i and $P(R_i = r_{ik}|Y = y_k)$ the probability that classifier i assigns rank r_{ik} to a sample k belonging to class $y_k \in \{0, 1\}$, which we simply denote as $P(R_i|y_k)$. Similarly, let $P(R_1, R_2, \cdots, R_M|y_k)$ denote the joint distribution that classifiers $1, \cdots, M$ assign ranks $R_1, \cdots, R_M$ to a given sample k belonging to class y_k. Further, we assume that N_1 of the total samples belong to the positive class as such let $\rho := N_1/N$ denote the prevalence of the positive class in the data.

In the current manuscript we are interested in linear ensembles, which is a weighted linear combination of individual classifier predictions. We next define the ensemble score (for a linear ensemble) assigned to sample k given a set of classifiers.

Definition 1. *Given a set of weights $w_i|_{i=1}^M$, let the random variable $S^{\mathbf{w}}$ denote the score assigned by the linear ensemble of classifiers $g_i|_{i=1}^M$. We can write $S^{\mathbf{w}}$ as follows:*

$$S^{\mathbf{w}} = \sum_{i=1}^{M} w_i R_i.$$

Hence, the ensemble score assigned to sample k is given as

$$S_k^{\mathbf{w}} = \sum_{i=1}^{M} w_i r_{ik}. \tag{1}$$

Using Definition 1, we want to first calculate the signal to noise score for the ensemble classifier. To do so we need to calculate the conditional mean and variance for ensemble score $S^{\mathbf{w}}$. The mean ensemble score conditioned on $Y = y$ is

$$\begin{aligned}
\mu_{ens|y} &= \mathbb{E}\left[S^{\mathbf{w}}|y\right] = \mathbb{E}\left[\sum_{i=1}^{M} w_i R_i \middle| y\right] \\
&= \sum_{i=1}^{M} w_i \mathbb{E}\left[R_i|y\right] \\
&= \sum_{i=1}^{M} w_i \mu_{i|y}
\end{aligned} \tag{2}$$

Similarly, since the ensemble score is a linear combination of random variables R_i, its conditional variance is given by

$$\sigma^2_{ens|y} = \mathbf{w}^T\,\mathbf{C}_y\,\mathbf{w}, \tag{3}$$

where $\mathbf{C}_y$ is the conditional covariance matrix whose (i, j)th value is given as

$$C_y(i, j) = \mathbb{E}\left[R_i R_j|y\right] - \mathbb{E}\left[R_i|y\right]\mathbb{E}\left[R_j|y\right].$$

Using equations (2) and (3), we can write the signal to noise score for the ensemble as

$$\begin{aligned} S^{\mathbf{w}} &:= \frac{\mu_{ens|0} - \mu_{ens|1}}{\sqrt{\sigma^2_{ens|0} + \sigma^2_{ens|1}}} = \frac{\sum_{i=1}^{M} w_i(\mu_{i|0} - \mu_{i|1})}{\sqrt{\mathbf{w}^T\,\mathbf{C}_0\,\mathbf{w} + \mathbf{w}^T\,\mathbf{C}_1\,\mathbf{w}}} \\ &= \frac{\sum_{i=1}^{M} w_i \Delta_i}{\sqrt{\mathbf{w}^T\,\mathbf{C}_0\,\mathbf{w} + \mathbf{w}^T\,\mathbf{C}_1\,\mathbf{w}}} \\ &= \frac{\boldsymbol{\Delta}^T\,\mathbf{w}}{\sqrt{\mathbf{w}^T\,\mathbf{C}\,\mathbf{w}}}, \end{aligned} \tag{4}$$

where $\boldsymbol{\Delta}, \mathbf{w} \in \mathbb{R}^M$ and $\mathbf{C} \in \mathbb{R}^{M \times M}$ are defined as

$$\Delta(i) := \mu_{i|0} - \mu_{i|1} \tag{5}$$

$$\mathbf{w}(i) := w_i \tag{6}$$

$$\mathbf{C} := \mathbf{C}_0 + \mathbf{C}_1 \tag{7}$$

Supplementary Note 2 MOCA weights

The MOCA ensemble is the optimal linear combination of base classifiers with classifier weights maximizing the ensemble signal to noise score given in (4). Before we prove this result, note that multiplying a set of classifier weights w_i by a constant c does not change the signal to noise score of the ensemble. Therefore, to find the MOCA weights, denoted as $\mathbf{w}^{\text{MOCA}}$, we will solve the following optimization problem:

$$\mathbf{w}^{\text{MOCA}} = \text{argmin}_{\mathbf{w}} \frac{\mathbf{w}^T \, \boldsymbol{\Delta}}{\sqrt{\mathbf{w}^T \, \mathbf{C} \, \mathbf{w}}} \quad \text{s.t.} \;\; \|\mathbf{w}\|_2 = 1, \tag{8}$$

where $\|\mathbf{w}\|_2$ represent the euclidean norm of the vector $\mathbf{w}$.

Theorem 1. *The maximizer of the optimization problem in* (8) *is given as*

$$\mathbf{w}^{MOCA} = \frac{\mathbf{C}^{-1} \, \boldsymbol{\Delta}}{\left\|\mathbf{C}^{-1} \, \boldsymbol{\Delta}\right\|_2}. \tag{9}$$

Proof. First note that the matrix $\mathbf{C}$ is a symmetric matrix and in this paper we assume that the classifiers are different and the conditional covariance matrices $\mathbf{C}_0$ and $\mathbf{C}_1$ are positive definite. As a result, the matrix $\mathbf{C}$ is positive definite and invertable. Hence, it has a Cholesky decomposition of the following form for an upper triangular matrix $\mathbf{U}$ [2]:

$$\mathbf{C} = \mathbf{U} \, \mathbf{U}^T \implies \mathbf{C}^{-1} = (\mathbf{U}^T)^{-1} \, \mathbf{U}^{-1} = (\mathbf{U}^{-1})^T \, \mathbf{U}^{-1}, \tag{10}$$

where we used the fact that $(\mathbf{U}^T)^{-1} = (\mathbf{U}^{-1})^T$. Next, let us calculate the signal to noise score of the ensemble with $\mathbf{w}^{\text{MOCA}} = \frac{\mathbf{C}^{-1} \, \boldsymbol{\Delta}}{\left\|\mathbf{C}^{-1} \, \boldsymbol{\Delta}\right\|_2}$. First, observe that $S^{\mathbf{w}}$, for $\mathbf{w}^{\text{MOCA}} = \frac{\mathbf{C}^{-1} \, \boldsymbol{\Delta}}{\left\|\mathbf{C}^{-1} \, \boldsymbol{\Delta}\right\|_2}$ is the same as when we let $\mathbf{w} = \mathbf{C}^{-1} \, \boldsymbol{\Delta}$ since $\left\|\mathbf{C}^{-1} \, \boldsymbol{\Delta}\right\|_2$ is constant. Hence, $S^{\mathbf{w}^{\text{MOCA}}}$ can be calculated as

$$\begin{aligned} S^{\mathbf{w}^{\text{MOCA}}} &= \frac{(\mathbf{C}^{-1} \, \boldsymbol{\Delta})^T \, \boldsymbol{\Delta}}{\sqrt{(\mathbf{C}^{-1} \, \boldsymbol{\Delta})^T \, \mathbf{C} \, \mathbf{C}^{-1} \, \boldsymbol{\Delta}}} \\ &= \frac{\boldsymbol{\Delta}^T (\mathbf{C}^{-1})^T \, \boldsymbol{\Delta}}{\sqrt{\boldsymbol{\Delta}^T (\mathbf{C}^{-1})^T \, \mathbf{C} \, \mathbf{C}^{-1} \, \boldsymbol{\Delta}}} \\ &= \sqrt{\boldsymbol{\Delta}^T \, \mathbf{C}^{-1} \, \boldsymbol{\Delta}} = \sqrt{\boldsymbol{\Delta}^T (\mathbf{U}^{-1})^T \, \mathbf{U}^{-1} \, \boldsymbol{\Delta}} = \left\|\mathbf{U}^{-1} \, \boldsymbol{\Delta}\right\|_2 . \end{aligned} \tag{11}$$

Let $\mathbf{w} \in \mathbb{R}^M$ be any set of classifier weights and since the matrix $\mathbf{U}^T$ is invertable there exists $\mathbf{w}_1$ such that

$$\mathbf{w} = (\mathbf{U}^T)^{-1} \, \mathbf{w}_1 .$$

Next, observe that

$$\begin{aligned} S^{\mathbf{w}} &= \frac{\mathbf{w}^T \, \boldsymbol{\Delta}}{\sqrt{\mathbf{w}^T \, \mathbf{C} \, \mathbf{w}}} = \frac{\mathbf{w}^T \, \boldsymbol{\Delta}}{\sqrt{\mathbf{w}^T \, \mathbf{U} \, \mathbf{U}^T \, \mathbf{w}}} = \frac{((\mathbf{U}^T)^{-1} \, \mathbf{w}_1)^T \, \boldsymbol{\Delta}}{\sqrt{((\mathbf{U}^T)^{-1} \, \mathbf{w}_1)^T \, \mathbf{U} \, \mathbf{U}^T ((\mathbf{U}^T)^{-1} \, \mathbf{w}_1)}} \\ &= \frac{\mathbf{w}_1^T \, \mathbf{U}^{-1} \, \boldsymbol{\Delta}}{\|\mathbf{w}_1\|_2} \leq \frac{\|\mathbf{w}_1\|_2 \left\|\mathbf{U}^{-1} \, \boldsymbol{\Delta}\right\|_2}{\|\mathbf{w}_1\|_2} = \left\|\mathbf{U}^{-1} \, \boldsymbol{\Delta}\right\|_2 , \end{aligned} \tag{12}$$

where in (12) we used the Cauchy-Schwarz inequality [2], which claims that for any two vector $\mathbf{u}$ and $\mathbf{v}$, the following inequality holds:

$$\mathbf{u}^T \mathbf{v} \leq \|\mathbf{u}\|_2 \, \|\mathbf{v}\|_2 .$$

Equations (11) and (12) together implies that for any $\mathbf{w}$

$$S^{\mathbf{w}} \leq S^{\mathbf{w}^{\mathrm{MOCA}}}.$$

Therefore, $\mathbf{w}^{\mathrm{MOCA}}$ maximizes $S^{\mathbf{w}}$ which completes the proof. $\square$

Supplementary Note 3 Some Intuition Behind Class-Conditioned Dependence between Classifier Predictions

To gain intuition on the meaning of class-conditioned independence of rank predictions, we show several possible cases using simulated data in Figure 1. Consider the ranking of $N = 500$ samples by the ith and j^{th} base classifiers shown in Figures 1A-D, where each sample is represented by a point whose x and y coordinates are the ranking r_i and r_j given to that sample by classifier i and j $(i \neq j)$ respectively. The fraction of positive class (orange) samples is $\rho = 0.4$. The conditional covariance matrix of rank predictions given class y, where $y = 0$ or $y = 1$, will be denoted by $\mathbf{C_y}$, and its element i, j is defined as $C_{y,ij} = \mathbb{E}(r_i r_j|y) - \mathbb{E}(r_i|y)\mathbb{E}(r_j|y)$. The element i, j of the (unconditioned) covariance matrix of rank predictions $\mathbf{Q}$ is given by $Q_{ij} = \mathbb{E}(r_i r_j) - \mathbb{E}(r_i)\mathbb{E}(r_j)$. Note that $\mathbb{E}(r_i) = \mathbb{E}(r_j) = (N + 1)/2$. because each of the N samples gets assigned a unique rank with no ties by each classifier. The figures also show the difference of the conditional means for each classifier, $\Delta_i = \mathbb{E}(r_i|0) - \mathbb{E}(r_i|1)$ and $\Delta_j = \mathbb{E}(r_i|0) - \mathbb{E}(r_i|1)$. Figures 1A,B show the case for which the rank assigned to a sample by one of the classifiers is independent of the rank assigned to the same sample by the other classifier, provided we consider samples within the same class (conditional independence given the class). Therefore in these cases $C_{0,ij} = C_{1,ij} = 0$. In Figure 1A, both Δ_i and Δ_j are positive, and in consequence both classifiers tend to predict class 1 samples in the lower ranks and class 0 samples in the higher ranks. This produces a positive association between the ranks of both classifiers, which results in a positive unconditioned covariance $Q_{ij} > 0$. The case of Figure 1B is slightly different, as classifier j is non-informative, with $\Delta_j = 0$. Therefore, even if $\Delta_i > 0$, in this case $Q_{ij} = 0$. It follows that if the rank predictions are conditionally independent given both classes and both classifiers are predictive $(\Delta > 0)$, then there is a positive correlation between the ranks assigned to the samples. However, if either one or both classifiers are uninformative, the unconditioned rank correlation is 0, as can be seen from Figure 1B for $\Delta_j = 0$. Indeed it can be proved [1] that under the assumption of class conditioned independent rank predictions, it is $Q_{ij} = \rho(1 - \rho)\Delta_i\Delta_j$.

When the rank predictions by classifiers are conditionally dependent (Figures 1C,D), the dependence between pairwise predictions r_i and r_j may lie on a diffuse clouds around the line $y = x$ (Figures 1C) or it may have have more complex structure (Figures 1D). Hence, the unconditioned covariance is typically non-zero regardless of the accuracy of the base classifiers as measured by $\Delta_{i,j}$ as it also carries information about the class-conditioned dependence. Indeed, it can be shown that the covariance of rank predictions can be written as $Q_{ij} = (1 - \rho)C_{0,ij} + \rho C_{1,ij} + \rho(1 - \rho)\Delta_i\Delta_j$.

Supplementary Note 4 Estimating MOCA weights for Unsupervised Ensemble Learning

In this section, we present a novel way to infer $\mathbf{w}^{\text{MOCA}}$, defined in Theorem 1, from unlabeled data. MOCA weights depends both on $\mathbf{\Delta}$ and the covariance matrix $\mathbf{C}$. Ahsen et al. [1] found that for base classifier with conditionally independent rank predictions, $\mathbf{\Delta}$ and ρ can be inferred from their covariance matrix and third order central moment tensor $T \in \mathbb{R}^{M\times M\times M}$ defined as

$$T(i,j,l) := \mathbb{E}\left[\Big(R_i - \mathbb{E}[R_i]\Big)\Big(R_j - \mathbb{E}[R_j]\Big)\Big(R_l - \mathbb{E}[R_l]\Big)\right]. \tag{13}$$

Similar to [1], we assume that the classifiers are conditionally independent in which case the off-diagonal entries of the matrix $\mathbf{C}$ is zero and we need to only infer the diagonals of it. The main result of this section shows, under the assumption of conditional independence, that the diagonal elements of $\mathbf{C}$ may be inferred from elements of $T(i,j,j)$ for $i \neq j$. Before we present this result, we need to define some notation.

Definition 2. *Given the rank ordered predictions of the ith base classifier, let*

$$\delta_i = \sigma^2_{i|0} - \sigma^2_{i|1}, \tag{14}$$

where $\sigma^2_{i|y} = \mathbb{E}\left[(R_i - \mu_{i|y})^2|y\right]$ for $y \in \{0,1\}$ is the conditional variance of the classifier i.

Next, let us formally define the conditional independence assumption.
Assumption 1 **(Conditional Independence of Classifiers):** The classifiers $g_i|_{i=1}^M$ are conditionally independent if

$$P(R_1, R_2, \ldots, R_M|Y=y) = \prod_{i=1}^M P(R_i|Y=y). \tag{15}$$

We need a final definition before proceeding with our analysis. For each classifier i, let

$$\mu_i := E[R_i] = \frac{N+1}{2},$$

which holds since each classifier assigns a unique rank in $[1,N]$ to each sample.

Lemma 1. *Assume Assumption* 1 *holds, then we have*

$$\mathbb{E}\left[(R_i-\mu_i)^2|Y=y\right] = \begin{cases} \sigma^2_{i|0} + \rho^2\Delta_i^2 & \text{if } y=0 \\ \sigma^2_{i|1} + (1-\rho)^2\Delta_i^2 & \text{if } y=1. \end{cases} \tag{16}$$

Proof. First note from the law of total expectation, we have

$$\mu_i = \mathbb{E}[R_i] = \rho\,\mathbb{E}[R_i|1] + (1-\rho)\,\mathbb{E}[R_i|0] = \rho\mu_{i|1} + (1-\rho)\mu_{i|0}, \tag{17}$$

and by the definition of Δ_i, we have

$$\Delta_i = \mu_{i|0} - \mu_{i|1}. \tag{18}$$

Combining (17) and (18), we find that

$$\mu_{i|y} = \begin{cases} \mu_i + \rho\Delta_i & \text{when } y=0 \\ \mu_i - (1-\rho)\Delta_i & \text{when } y=1 \end{cases} \tag{19}$$

Next, observe that

$$\begin{aligned}
\mathbb{E}\left[(R_i-\mu_i)^2|Y=y\right] &= \mathbb{E}\left[R_i^2|Y=y\right]-2\mu_i\mathbb{E}\left[R_i|Y=y\right]+\mu_i^2 \\
&= \mathbb{E}\left[R_i^2|Y=y\right]-\mu_{i|y}^2+\mu_{i|y}^2-2\mu_i\mathbb{E}\left[R_i|Y=y\right]+\mu_i^2 \\
&= \sigma_{i|y}^2+\mu_{i|y}^2-2\mu_i\mu_{i|y}+\mu_i^2 \\
&= \sigma_{i|y}^2+(\mu_{i|y}-\mu_i)^2. \qquad (20)
\end{aligned}$$

If we put (19) into (20), we obtain

$$\mathbb{E}\left[(R_i-\mu_i)^2|Y=y\right]=\begin{cases}\sigma_{i|0}^2+\rho^2\Delta_i^2 & \text{if } y=0\\ \sigma_{i|1}^2+(1-\rho)^2\Delta_i^2 & \text{if } y=1,\end{cases}$$

which completes the proof of the theorem. □

Next theorem gives a closed form formula for $C(i,j)$ in terms of δ_i.

Theorem 2. *Under the conditional independence assumption, the elements of matrix* $\mathbf{C}$*,* $\mathbf{C}(i,j)$*, is given as*

$$C(i,j)=\begin{cases}0 & \text{if } i\neq j,\\ (N^2-1)/6+(2\rho-1)\delta_i-2\rho(1-\rho)\Delta_i^2 & \text{if } i=j.\end{cases}$$

Proof. First observe that, under the conditional independence assumption, for $i\neq j$, $C_0(i,j)=C_1(i,j)=0$. Hence, $C(i,j)=C_0(i,j)+C_1(i,j)=0$. Next, let us calculate diagonal entries of $\mathbf{C}$. From the definition of conditional covariance for $i=j$, we have

$$C(i,i)=C_0(i,i)+C_1(i,i)=\sigma_{i|0}^2+\sigma_{i|1}^2.$$

By the law of total expectation for any i, we have

$$\begin{aligned}
\mathrm{Var}(R_i) &= \rho\mathbb{E}\left[(R_i-\mu_i)^2|Y=1\right]+(1-\rho)\mathbb{E}\left[(R_i-\mu_i)^2|Y=0\right] \\
&= \rho\sigma_{i|1}^2+(1-\rho)\sigma_{i|0}^2+\rho(1-\rho)\Delta_i^2 \qquad (21)\\
&= -\rho(\sigma_{i|0}^2-\sigma_{i|1}^2)+\sigma_{i|0}^2+\rho(1-\rho)\Delta_i^2 \\
&= -\rho(\sigma_{i|0}^2-\sigma_{i|1}^2)+\frac{1}{2}\left(\sigma_{i|0}^2+\sigma_{i|0}^2+\sigma_{i|1}^2-\sigma_{i|1}^2\right)+\rho(1-\rho)\Delta_i^2 \\
&= \left(\frac{1}{2}-\rho\right)(\sigma_{i|0}^2-\sigma_{i|1}^2)+\frac{1}{2}\left(\sigma_{i|0}^2+\sigma_{i|1}^2\right)+\rho(1-\rho)\Delta_i^2 \\
&= \left(\frac{1}{2}-\rho\right)\delta_i+\frac{1}{2}C(i,i)+\rho(1-\rho)\Delta_i^2, \qquad (22)
\end{aligned}$$

where in deriving (21), we used (16) from Lemma 1. Note that all classifiers assign a unique rank, in the interval $[1,N]$, to each of the N samples. Therefore, the variance of R_i is that of the discrete uniform distribution, $\mathrm{Var}(R_i)=(N^2-1)/12$. Rearranging the terms in Equation (22) gives us the desired result that

$$C(i,i)=(N^2-1)/6+(2\rho-1)\delta_i-2\rho(1-\rho)\Delta_i^2.$$

□

Our final result gives a closed form formula for each element of the covariance tensor T in terms of δ_i.

Theorem 3. *Given M conditionally independent sample rank predictions by the set of base classifiers $\{g_i\}_{i=1}^{M}$, the elements of the third central moment tensor T_{ijj} for $i \neq j$ are*

$$T(i,j,j) = \rho(1-\rho)\Delta_i \Big(\delta_j + (2\rho - 1)\Delta_j^2.\Big)$$

Proof. For $i \neq j$, the elements of the third central moment tensor T are given

$$T(i,j,j) = \mathbb{E}\left[(R_i - \mu_i)(R_j - \mu_j)^2\right]. \tag{23}$$

Applying the law of total expectation to (23) for conditionally independent classifier predictions result in

$$T(i,j,j) = \rho\mathbb{E}\left[(R_i - \mu_i)|1\right]\mathbb{E}\left[(R_j - \mu_j)^2|1\right] + (1-\rho)\mathbb{E}\left[(R_i - \mu_i)|0\right]\mathbb{E}\left[(R_j - \mu_j)^2|0\right]. \tag{24}$$

Then substituting equations (16) and (19) into (24), we find that

$$T(i,j,j) = \rho(1-\rho)\Delta_i\left[\sigma_{j|0}^2 - \sigma_{j|1}^2 + (2\rho - 1)\Delta_j^2\right] = \rho(1-\rho)\Delta_i\left[\delta_j + (2\rho-1)\Delta_j^2\right]. \tag{25}$$

□

Using the above result, we can calculate $T(i,j,j)$ for $i, j \in \{1, 2, \ldots, M\}$ from the rank predictions of each base classifier without the use of labels. Using the algorithm present in [1], we can estimate ρ, Δ_i in an unsupervised manner, which can be used to get an estimate for δ_i using Theorem 3. Finally using the estimates for ρ, Δ_i and δ_i, we can estimate $C(i,i)$ using Theorem 2.

Theorem 3 indicates that we can use any $i \neq j$ to calculate δ_j. However, in practical applications the measurements are noisy and it is more robust to estimate δ_j by linear regression. We do so by defining

$$\alpha_j := \frac{\delta_j}{\|\boldsymbol{\Delta}\|_2}$$

and infer α_j by linear regression

$$\hat{\alpha}_j = \text{argmin}_{\alpha_j} \sum_{i \neq j} \left(\frac{T(i,j,j) - \lambda_t \nu_i \nu_j^2}{\lambda_c} - \alpha_j \nu_i\right)^2, \tag{26}$$

where $\lambda_c := \rho(1-\rho)\|\boldsymbol{\Delta}\|_2^2$, $\lambda_t := \rho(1-\rho)(2\rho-1)\|\boldsymbol{\Delta}\|_2^3$, and $\nu_i = \Delta_i / \|\boldsymbol{\Delta}\|_2$ as defined by Ahsen et al. [1]. We then infer $C(i,i)$ by substituting $\delta_j = \|\boldsymbol{\Delta}\|_2 \alpha_j$ into Theorem 2.

Supplementary Note 5 Comparison of uMOCA to other unsupervised ensemble methods

uMOCA is an unsupervised classification ensemble method rooted in a theoretical framework which makes some assumptions about the correlation relationship between base classifiers. Under those assumptions, uMOCA offers some flexibility in accommodating base classifier predictions without any distributional assumptions, which makes it a promising algorithm for a wide range of unsupervised learning tasks. Unsupervised ensemble learning can also be done with unsupervised clustering algorithms. One such example is GMM (Gaussian Mixture Models), a classic method in the field of clustering, that operates on the assumption that data points are generated from a mixture of Gaussian distributions. With its well-established theoretical foundation and intuitive interpretation, GMM has been extensively used in practice, particularly when data conform to Gaussian assumptions.

In this section, we compare the performance of uMOCA, GMMs and the WOC method, employing a variety of datasets. Similar to Figure 3 of the main text, we use four datasets for this purpose. We use two simulated datasets: one with conditionally uncorrelated base-classifiers predictions (Supplementary Figure 2A) and the other with conditionally correlated base classifiers predictions (Supplementary Figure 2C). We also use the DREAM2 BCL6 Transcription Factor Target prediction challenge (Supplementary Figure 2B) and the DREAM9.5 Prostate Cancer Survival prediction challenge (Supplementary Figure 2D). We observe that when the conditional correlation between base classifiers is weak (see Supplementary Figures 2)A and B), uMOCA significantly outperforms the GMM method showing a degree of robustness of uMOCA in unsupervised learning problems, especially when the individual classifiers are nearly conditionally independent, where our theory shows that uMOCA is the optimal linear ensemble classifier. When the classifiers are not conditionally independent, then our results are mixed. The GMM algorithm performance is better uMOCA in simulated data with conditionally dependent classifiers, whereas both uMOCA and GMM perform similar in the DREAM9.5 Prostate Cancer Survival prediction challange. This illustrates that in the case of conditionally correlated base classifiers uMOCA does not have a distinctive advantage over GMM.

Figures

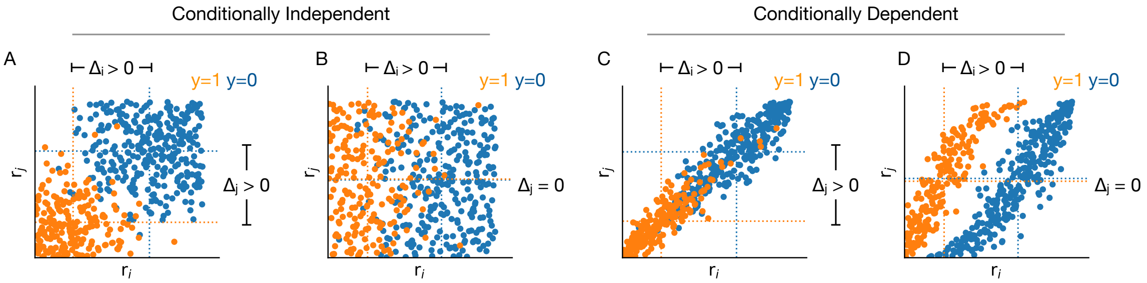

Supplementary Figure 1: **Pairwise comparison of rank ordered base classifier predictions.** The empirical covariance (Q_{ij}) between the rank ordered predictions by methods i and j is determined by their respective performance (Δ_i, Δ_j) and their conditional correlation (C_{ij}). Figures A-D show simulated rank predictions of 500 samples in which 200 samples (prevalence ρ=0.4) are from the positive class (y=1). In (A) AUC$_i$ = AUC$_j$ = 0.95 and $C_{ij} = 0$ resulting in $Q_{ij} > 0$, (B) AUC$_i$ = 0.95, AUC$_j$ = 0 and $C_{ij} = 0$ resulting in $Q_{ij} = 0$, (C) AUC$_i$ = AUC$_j$ = 0.95 and $C_{ij} = 0.9$ resulting in $Q_{ij} > 0$, (D) AUC$_i$ = 0.95, AUC$_j$ = 0 and $C_{ij} = 0.9$ resulting in $Q_{ij} > 0$.

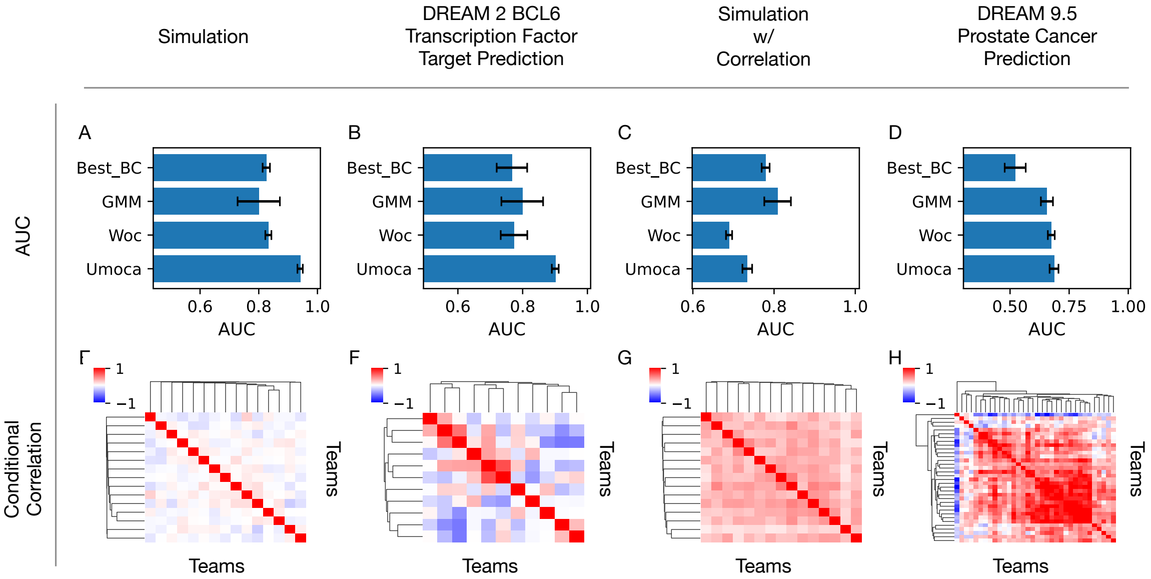

Supplementary Figure 2: **Comparison of uMOCA with other unsupervised models.** Classifiers were applied to simulation data where base classifier predictions are conditionally independent (A,E), DREAM BCL6 Transcription factor target prediction (B,F), simulation data where base classifier predictions are conditionally dependent (C,G), and DREAM 9.5 Prostate cancer survival prediction (D,H). For each data set we measure the AUC in relation to uMOCA, wisdom of crowd ensemble (WOC), the best individual base classifier (Best_BC), and Gaussian Mixture Model (GMM) implemented in scikit-learn assuming full covariance matrices; and measure the empirical conditional covariance matrix C. The error-bars represent S.E.M. computed from 5 fold cross-validation.

References

1. Ahsen, M. E., Vogel, R. M. & Stolovitzky, G. A. Unsupervised Evaluation and Weighted Aggregation of Ranked Classification Predictions. *Journal of Machine Learning Research* **20,** 1–40 (2019).
2. Horn, R. A. & Johnson, C. R. *Matrix analysis* (Cambridge university press, 2012).
